# Supplementary material for: Exploring Professional Practice Environments and Organisational Context Factors Affecting Nurses’ Adoption of Evidence-Based Practice: A Scoping Review
Source: Healthcare (Basel). 2024 Jan 18;12(2):245. doi: 10.3390/healthcare12020245 (PMC10815808; doi:10.3390/healthcare12020245)
Supplement: Supplementary file 1 [file healthcare-12-00245-s001.zip › Table_S3.pdf]

Table S3. Search strategy conducted in the PubMed, Web of Science, CINAHL (via EBSCO) and MEDLINE (via EBSCO) databases, on 1 March 2023.

SEARCH NO.	SEARCH TERMS AND EXPRESSIONS	RESULTS
	PubMed	
#1	"Nursing"[Mesh] OR "Nurses"[Mesh] OR "nurs*"[tiab]	674.115
#2	"Students"[Mesh] OR "nursing student*"[tiab] OR "student*"[tiab]	388.165
#3	#1 NOT #2	623.613
#4	"facilitator"[tiab] OR "enabler"[tiab] OR "enhancer"[tiab] OR "implement*"[tiab] OR "helper"[tiab]	785.792
#5	"hindering"[tiab] OR "obstacle"[tiab] OR "barrier"[tiab] OR "difficult*"[tiab] OR "impediment*"[tiab]	1.021.015
#6	#4 OR #5	1.756.602
#7	"Evidence-Based Practice"[Mesh] OR "evidence-based practice"[tiab] OR "EBP"[tiab]	107.748
#8	#6 AND #7	20.032
#9	"health organization*"[tiab] OR "healthcare organization*"[tiab] OR "healthcare organization*"[tiab] "hospital*l"[tiab] OR "organization*"[tiab] OR "organization*"[tiab]	460.317
#10	#3 AND #8 AND #9	358
	Web of Science	
#1	((TS=(Nursing)) OR TS=(Nurses)) OR TS=(nurs*)	410.048
#2	((TS=(Students)) OR TS=(nursing student*)) OR TS=(student*)	1.102.549
#3	#1 NOT #2	370.057
#4	((((TS=(facilitator)) OR TS=(enabler)) OR TS=(enhancer)) OR TS=(implement*)) OR TS=(helper)	2.519.232
#5	((((TS=(hindering)) OR TS=(obstacle)) OR TS=(barrier)) OR TS=(difficult*)) OR TS=(impediment)	2.508.367
#6	#4 OR #5	4.792.566
#7	(TS=(Evidence-Based Practice)) OR TS=(EBP)	83.096
#8	#6 AND #7	31.434
#9	(((((TS=(health organization)) OR TS=(healthcare organization*)) OR TS=(healthcare organization*)) OR TS=(hospital*)) OR TS=(organization*)) OR TS=(organization*)	2.484.535
#10	#3 AND #8 AND #9	1.455
	CINAHL	
S1	MH "Nurses" OR TI "Nurs*" OR AB "Nurs*"	652.048
S2	MH "Students, College" OR MH "Students, Nursing" OR MH "Students, Pre-Nursing" OR TI "Undergraduat*" OR AB "Undergraduat*" OR TI "Student*" OR AB "Student*" OR TI "nursing student*" OR AB "nursing student*"	237.966
S3	S1 NOT S2	591.037
S4	AB "facilitator" OR AB "enabler" OR AB "enhancer" OR AB "implement*" OR AB "helper"	234.919
S5	AB "hindering" OR AB "obstacle" OR AB "barrier" OR AB "difficult*" OR AB "impediment*"	208.384
S6	S4 OR S5	426.094
S7	MH "Professional Practice, Evidence-Based" OR TI "evidence-based practice" OR AB "evidence-based practice" OR TI "EBP" OR AB "EBP"	39.241
S8	S6 AND S7	7.442

S9	AB “health organization*” OR AB “healthcare organization*” OR AB “healthcare organization*” OR AB “hospital*” OR AB “organization*” OR AB “organization*”	590.43
S10	S3 AND S8 AND S9	623
	MEDLINE	
S1	MH "Nurses" OR TI “Nurs*” OR AB “Nurs*”	526.266
S2	MH "Students, College" OR MH "Students, Nursing" OR MH "Students, Pre-Nursing" OR TI “Undergraduat*” OR AB “Undergraduat*” OR TI “Student*” OR AB “Student*” OR TI “nursing student*” OR AB “nursing student*”	377.263
S3	S1 NOT S2	479.521
S4	AB "facilitator" OR AB “enabler” OR AB “enhancer” OR AB “implement*” OR AB “helper”	744.469
S5	AB “hindering” OR AB “obstacle” OR AB “barrier” OR AB “difficult*” OR AB “impediment*”	981.126
S6	S4 OR S5	1.677.935
S7	MH "Professional Practice, Evidence-Based" OR TI “evidence-based practice” OR AB “evidence-based practice” OR TI “EBP” OR AB “EBP”	19.246
S8	S6 AND S7	6.718
S9	AB “health organization*” OR AB “healthcare organization*” OR AB “healthcare organization*” OR AB “hospital*” OR AB “organization*” OR AB “organization*”	1.771.592
S10	S3 AND S8 AND S9	453
TOTAL		
