# Supplementary material for: Exploring Professional Practice Environments and Organisational Context Factors Affecting Nurses’ Adoption of Evidence-Based Practice: A Scoping Review
Source: Healthcare (Basel). 2024 Jan 18;12(2):245. doi: 10.3390/healthcare12020245 (PMC10815808; doi:10.3390/healthcare12020245)
Supplement: Supplementary file 1 [file healthcare-12-00245-s001.zip › Table_S4.pdf]

Table S4. Overview of the characteristics of the primary studies included in the scoping review.

Author(s)	Source	Title	Study type	Participants	Aim(s)
Al-Maskari el al. (2018) [60], Oman	Sultan Qaboos University Medical Journal	Attitudes towards and perceptions regarding the implementation of Evidence-Based Practice among Omani nurses	Cross-sectional study	98 nurse managers<br>162 staff nurses	To explore Omani nurses' attitudes towards EBP implementation and their perceptions of barriers and facilitators to EBP implementation.<br>To compare differences in the attitudes and perceptions of Omani nurse managers and clinical staff nurses, considering that these two professional groups play key roles in providing high-quality nursing care.
Fry et al. (2018) [57], Australia	International Journal of Evidence-Based Healthcare	Nursing and midwifery use, perceptions and barriers to evidence-based practice: a cross-sectional survey	Cross-sectional study	204 staff nurses	Explore how evidence-based knowledge and evidence-based practice are obtained, utilised and practised in a clinical context.
Gifford et al. (2018) [55], China	BMC Nursing	When east meets west: a qualitative study of barriers and facilitators to evidence-based practice in Hunan China	Qualitative descriptive study	2 nurse managers<br>6 nurse managers<br>5 staff nurses	To explore barriers and facilitators to evidence-based practice in Hunan province, a less developed region in China.
van der Goot et al. (2018) [46], Netherlands	Nurse Education Today	The effect of a multifaceted evidence-based practice programme for nurses on knowledge, skills, attitudes, and perceived barriers: A cohort study	Cross-sectional study	58 staff nurses	To measure the results of a six-month EBP programme on nurses' knowledge, skills, attitudes and perception of barriers in a context of implementing EBP.
Azmoude et al. (2018) [42], Iran	Malaysian Journal of Medical Sciences	Midwives' attitude and barriers of Evidence Based Practice in maternity care	Cross-sectional study	76 midwife nurses	To describe the attitude of Iranian obstetric nurses and their perception of the barriers to evidence-based practice in the context of maternal and obstetric health nursing care.
Nkrumah et al. (2018) [47], Ghana	Pan African Medical Journal	Barriers for nurses' participation in and utilisation of clinical research in three hospitals within the Kumasi Metropolis, Ghana	Cross-sectional study	158 staff nurses	To study the proportion of nurses involved in clinical research activities, as well as the barriers to the implementation of EBP by nurses practising in health institutions in the Kumasi metropolitan area in Ghana.
Shuman et al. (2018) [63], USA	Implementation Science	Associations among unit leadership and unit climates for implementation in acute care: a cross-sectional study	Cross-sectional study	22 nurse managers<br>287 staff nurses	To analyse the contribution of nurse managers in terms of leadership and competence in EBP in what shapes the organisational climate of medical-surgical services in the implementation of EBP.

Lafuente-Lafuente et al. (2019) [68], France	BMJ Open	Knowledge and use of evidence-based medicine in daily practice by health professionals: a cross-sectional survey	Cross-sectional study	54 staff nurses	To understand the regularity with which health professionals have actually used EBP in their daily clinical practice.
Pittman et al. (2019) [64], USA	Worldviews on Evidence-Based Nursing	A multisite health system survey to assess organizational context to support Evidence-Based Practice	Cross-sectional study	94 nurse managers 701 staff nurses	To study the organisational hospital context and the level of preparation for implementing EBP, and to describe EBP implementing in a hospital context.
Alqahtani et al. (2020) [9], Saudi Arabia	Journal of Clinical Nursing	Nurses' evidence-based practice knowledge, attitudes and implementation: A cross-sectional study	Cross-sectional study	227 staff nurses	To study the influence of nurses' individual factors in terms of knowledge, attitudes and implementation of EBP in Saudi Arabia and to identify facilitators and barriers to the implementation of EBP from the perspective of this professional group.
Chen et al. (2020) [38], China	Journal of Nursing Management	Value, knowledge and implementation on evidence-based practice among nurse managers in china: A regional cross-sectional survey	Cross-sectional study	1166 nurse managers	To describe the value, knowledge and implementation of evidence-based practice (EBP) among Chinese nurse managers, as well as the factors associated with their implementation of EBP.
Fu et al. (2020) [54], China	Nursing & Health Sciences	The barriers to evidence-based nursing implementation in mainland China: A qualitative content analysis	Qualitative descriptive study	45 staff nurses	To describe the participants' experiences and opinions on the barriers to the implementation of evidence-based nursing in mainland China.
Dessie et al. (2020) [52], Ethiopia	Current Therapeutic Research	Evidence-Based Practice and associated factors among health care providers working in public hospitals in Northwest Ethiopia during 2017	Cross-sectional study	342 staff nurses	To assess the reported level of EBP utilisation and the factors associated with its implementation among healthcare professionals working in public hospitals in north-west Ethiopia.
Ost et al. (2020) [28], USA	Critical Care Nurse	Aligning Organizational Culture and Infrastructure to Support Evidence-Based Practice	Project to promote EBP adoption	NA	Developing an integrated system to develop practices for implementing EBP in order to increase the results and gains of the people who receive nursing care.
Renolen et al. (2020) [32], Norway	Research in Nursing & Health	Creating room for evidence-based practice: Leader behavior in hospital wards	Grounded Theory	63 staff nurses	To develop a theory about the behavioural patterns of leaders who are involved in the integration and implementation of EBP in clinical practice contexts.
Yiridomoh et al. (2020) [66], Ghana	Rural Society	Evidence-based practice and rural health service delivery: knowledge and barriers to adoption among clinical nurses in Ghana	Qualitative descriptive study	13 staff nurses	To understand, explain and demystify nurses' experience and knowledge of using EBP.
Duff et al. (2020) [33], USA	Implementation Science Communications	Determinants of an evidence-based practice environment: an interpretive description	Interpretative description (qualitative)	12 staff nurses	To gather nurses' perspectives and experiences of using the Iowa Model of EBP in order to help inform its introduction in other clinical practice settings.

Alshammari et al. (2021) [44], Saudi Arabia	Nurse Media Journal of Nursing	Factors affecting the implementation and barriers to Evidence-Based Practice among nurse practitioners in Hail Region, Saudi Arabia	Cross-sectional study	34 nurse managers<br>194 staff nurses	To investigate the factors that affect the implementation of EBP and to determine the barriers to its implementation as perceived by nurses.
Dagne et al (2021) [30], Ethiopia	PLoS ONE	Implementation of evidence-based practice: The experience of nurses and midwives	Qualitative descriptive study	86 staff nurses	To explore the implementation of evidence-based practice by nurses and obstetric nurses practising in public hospitals.
Dagne et al. (2021) [67], Ethiopia	Reproductive Health	Implementation of evidence-based practice and associated factors among nurses and midwives working in Amhara Region government hospitals: a cross-sectional study	Cross-sectional study	77 nurse managers<br>254 midwife nurses<br>459 nurses	To evaluate the implementation of EBP and the factors associated with it among nurses and obstetric nurses.
Alqahtani et al. (2022) [45], Saude Arabia	Nursing Reports	Barriers to implementing Evidence-Based Practice among primary healthcare nurses in Saudi Arabia: A cross-sectional study	Cross-sectional study	93 nurse managers<br>191 nurses	To study the barriers perceived by primary health care nurses in relation to the implementation of EBP.
Alqahtani et al. (2022) [34], Saudi Arabia	International Journal of Environmental Research and Public Health	Organizational factors associated with Evidence-Based Practice knowledge, attitudes, and implementation among nurses in Saudi Arabia	Cross-sectional study	227 staff nurses	To examine the organisational factors that influence nurses' knowledge, attitudes and implementation of EBP and to identify their perceptions of nursing leadership in EBP and hospital support for its implementation in Saudi Arabia.
Crawford et al. (2022) [29], USA	Worldviews on Evidence-Based Nursing	Barriers and facilitators influencing EBP readiness: Building organizational and nurse capacity	Cross-sectional study	724 staff nurses	Describe EBP preparedness, barriers, and facilitators reported by nurses in a nationwide healthcare system before COVID-19.
Lanssens et al. (2022) [65], Belgium	European Journal of Midwifery	Knowledge, attitudes and use of evidence-based practice among midwives in Belgium: A cross-sectional survey	Cross-sectional study	251 midwife nurses	To explore the practice, attitudes and barriers associated with the implementation of EBP in the context of professional midwifery practice in hospitals and primary health care by nurse midwives in Flanders - Belgium.
Li et al. (2022) [58], China	International Journal of Nursing Sciences	Knowledge, attitude and behaviour to evidence-based practice among psychiatric nurses: A cross-sectional survey	Cross-sectional study	923 mental health and psychiatric nurses	To determine the level of knowledge, attitudes and behaviour towards evidence-based practice (EBP) among Chinese psychiatric nurses, and to study the factors that influence the implementation of EBP.

NA: not applicable.
