# Supplementary material for: Exploring Professional Practice Environments and Organisational Context Factors Affecting Nurses’ Adoption of Evidence-Based Practice: A Scoping Review
Source: Healthcare (Basel). 2024 Jan 18;12(2):245. doi: 10.3390/healthcare12020245 (PMC10815808; doi:10.3390/healthcare12020245)
Supplement: Supplementary file 1 [file healthcare-12-00245-s001.zip › Table_S5.pdf]

Table S5. Overview of the characteristics of the secondary studies included in the scoping review.

Author(s)	Source	Title	Study type	Included documents	Aim(s)
Mathieson et al (2018) [35], United Kingdom	Primary Health Care Research and Development	Strategies, facilitators and barriers to implementation of evidence-based practice in community nursing: a systematic mixed-studies review and qualitative synthesis	Mixed methods systematic review of the literature review with qualitative synthesis	22 documents	To evaluate and synthesise empirical literature on the implementation of evidence within community nursing, and to explore the use of implementation theory and identify the strategies needed and the barriers and facilitators to successful implementation within this context.
Bianchi et al. (2018) [62], Irland	Journal of Nursing Management	A review of the role of nurse leadership in promoting and sustaining evidence-based practice	Integrative review of the literature	28 documents	To explore how nursing leadership influences evidence-based practice in healthcare practice settings.
Shayan et al. (2019) [40], Uganda	Worldviews on Evidence-Based Nursing	Barriers associated with Evidence-Based Practice among nurses in low- and middle-income countries: A systematic review	Systematic review of the literature	16 documents	To study the barriers to the implementation of EBP among nurses working in low- and middle-income countries.
Shifaza et al. (2019) [53], Iran	Journal of Evidence-Based Care	System factors influencing the Australian nurses' evidence-based clinical decision making: A systematic review of recent studies	Systematic review of the literature	18 documents	To systematically review the literature on factors associated with health systems and their possible impact on the process of implementing evidence-based practice.
Lizarondo et al. (2019) [56], Australia	Worldviews on Evidence-Based Nursing	Barriers and facilitators to implementing evidence in African health care: A content analysis with implications for action	Review of the literature (documental analysis)	20 documents	To determine barriers and facilitators to the implementation of evidence in health contexts located in Africa, based on implementation projects carried out as part of the Joanna Briggs Institute (JBI) clinical fellowship programme.
Ayoubian et al. (2020) [39], Iran	Galen Medical Journal	Evaluation of facilitators and barriers to implementing Evidence-Based Practice in the health services: A systematic review	Systematic review of the literature	12 documents	Systematically review the main relevant articles on barriers and facilitators to the implementation of evidence-based practice in health services.
Clavijo-Chamorro et al. (2020) [51], Spain	Western Journal of Nursing Research	Context as a facilitator of the implementation of Evidence-based Nursing: A meta-synthesis	Qualitative systematic review of the literature with meta-synthesis	57 documents	To explore the factors that facilitate the implementation of scientific evidence in clinical nursing practice, related to the work context.

Nelson-Brantley et al. (2020) [37], USA	Journal of Nursing Administration	Magnet(R) and Pathway to Excellence(R) focusing on research and Evidence-Based Practice	Review of the literature	5 documents	Describe common barriers and effective strategies for developing a culture of research and evidence-based practice, with recommendations for accredited and non-accredited organisations.
Pitsillidou et al. (2020) [69], Cyprus	Journal of Nursing and Social Sciences Related to Health and Illness	Barriers to the adoption of evidence-based practice among nurses	Systematic review of the literature	14 documents	To study the barriers that prevent nurses from evaluating and applying evidence from research results in their nursing practice.
Younas (2020) [43], Canada	Nursing	Identifying international barriers and facilitators to research utilization	Integrative review of the literature	42 documents	Define the concept of research utilisation and identify barriers and facilitators to the use of research in clinical nursing practice.
Berthelsen et al. (2021) [50], Denmark	Worldviews on Evidence-Based Nursing	The importance of context and organization culture in the understanding of nurses' barriers against Research Utilization: A systematic review	Systematic review of the literature	27 documents	To identify nurses' perceptions of the barriers to the use of research in clinical practice between 2000 and 2018, on all continents, by reviewing studies that used the Barriers to Research Utilisation scale (BARRIERS).
McArthur, et al. (2021) [61], Canada	Implementation Science	Barriers and facilitators to implementing evidence-based guidelines in long-term care: a qualitative evidence synthesis	Qualitative systematic review of the literature	33 documents	Systematise the barriers and facilitators associated with implementing EBP in the context of long-term care.
Clavijo-Chamorro et al. (2022) [41], Spain	Western Journal of Nursing Research	Leadership as a facilitator of Evidence Implementation by nurse managers: A metasynthesis	Qualitative systematic review of the literature with meta-synthesis	11 documents	To clarify the factors that facilitate the implementation of scientific evidence by nurses, in the way it is perceived by nurse managers, considering their experience in terms of implementing research results.
Dakka (2022) [48], Israel	SAGE Open Nursing	Nurses barriers to Evidence-Based Practice in palliative care: A systematic review	Systematic review of the literature with meta-analysis	7 documents	To investigate nurses' barriers to implementing EBP in palliative care.
Teixeira et al. (2022) [36], Portugal	Journal of Clinical Nursing	Professional empowerment and evidence-based nursing: A mixed-method systematic review	Mixed methods systematic review of the literature	9 documents	To review, synthesise and integrate the results of primary studies on the relationship between professional empowerment and evidence-based practice in nurses.
Zhao et al. (2022) [59], China	The Lancet Regional Health Western Pacific	Evidence-based practice implementation in healthcare in China: a living scoping review	Scoping review	309 documents	To provide an overview of the progress of research into the implementation of EBP in China and to identify gaps for future studies.
